# Isolation and Screening of Microorganisms for the Effective Pretreatment of Lignocellulosic Agricultural Wastes

**DOI:** 10.1155/2021/5514745

**Published:** 2021-09-21

**Authors:** Zichen Zhang, Aabid Manzoor Shah, Hassan Mohamed, Nino Tsiklauri, Yuanda Song

**Affiliations:** ^1^Colin Ratledge Center for Microbial Lipids, School of Agriculture Engineering and Food Sciences, Shandong University of Technology, Shandong Zibo 255000, China; ^2^Normal College, Jishou University, Jishou, 416000 Hunan, China; ^3^Department of Botany and Microbiology, Faculty of Science, Al-Azhar University, Assiut 71524, Egypt; ^4^Durmishidze Institute of Biochemistry and Biotechnology, Academy of Sciences of Georgia, 10 km Agmashenebeli Alley, 0159 Tbilisi, Georgia

## Abstract

Lignocellulosic waste is the most abundant biorenewable biomass on earth, and its hydrolysis releases highly valued reducing sugars. However, the presence of lignin in the biopolymeric structure makes it highly resistant to solubilization thereby hindering the hydrolysis of cellulose and hemicellulose. Microorganisms are known for their potential complex enzymes that play a dominant role in lignocellulose conversion. Therefore, the current study was designed to isolate and screen potential microorganisms for their selective delignification ability for the pretreatment of lignocellulosic biomass. An extensive isolation and screening procedure yielded 36 desired isolates (22 bacteria, 7 basidiomycete fungi, and 7 filamentous fungi). Submerged cultivation of these desired microorganisms revealed 4 bacteria and 10 fungi with potent lignocellulolytic enzyme activities. The potent isolates were identified as *Pleurotus*, *Trichoderma*, *Talaromyces*, *Bacillus*, and *Chryseobacterium* spp. confirmed by morphological and molecular identification. The efficiency of these strains was determined through enzyme activities, and the degraded substrates were analyzed through scanning electron microscopy (SEM) and X-ray diffraction (XRD). Among all isolated microbes, *Pleurotus* spp. were found to have high laccase activity. The cellulose-decomposing and selective delignification strains were subjected to solid-state fermentation (SSF). SSF of field waste corn stalks as a single-carbon source provides *Pleurotus* spp. better condition for the secretion of ligninolytic enzymes. These isolated ligninolytic enzymes producing microorganisms may be used for the effective pretreatment of lignocellulosic agricultural wastes for the production of high value-added natural products by fermentation.

## 1. Introduction

Lignocellulosic biomass represents an important carbon-neutral renewable resource for the production of bioenergy and biomaterials [1]. Huge reserves of lignocellulose are agroforestry residues, crops, agrowastes, grass, and algae. Crystallized cellulose and hemicellulose polymer matrix are packed by the highly polymerized phenolic lignin in lignocellulosic biomass that leads to the difficulties in digestibility and availability of cellulosic and hemicellulosic fractions. Lignocellulose represents a considerable source of alternative energy that can minimize the rapid consumption of nonrenewable fossil resources like petroleum, natural gas, coal, and minerals. The gradual conversion of substantial sectors of the global economy to a sustainable biobased economy, with bioenergy, biofuels, and biobased products as its main pillars, is a forward-looking approach [2]. Biofuels derived from lignocellulosic materials are crucial for biorefineries because they can replace the importance of petrochemistry in modern society. Plant polysaccharides-lignin composite can be used to produce the majority of these sustainable biorefinery products [3]. The structural configuration of lignocellulose biomass is a complex that creates a reluctant nature for enzymatic hydrolysis [4, 5]. However, a pretreatment method is required to overcome the physical and chemical barriers present in the lignin–carbohydrate composite and make the majority of the plant cell wall components easily accessible for conversion into valuable products [6–8]. Because people are becoming more aware of their actions' impact on the environment, sustainability is gaining a lot of attention. To rethink and reinvent processes in such a way that materials and energy are used more effectively inside a closed-loop system, a paradigm shift is necessary [9]. Pretreatment is a critical step in establishing an economically viable biorefinery. A successful pretreatment method must result in partial or total separation of the lignocellulosic components, increasing cellulose accessibility to enzymatic hydrolysis and releasing the least inhibitory compounds for subsequent steps of enzymatic hydrolysis and fermentation. Each pretreatment technology has a different specificity against carbohydrates and lignin and may or may not be effective for different types of biomass [3]. Various pretreatment procedures for cellulosic resources have been developed, such as chemical processes (acid/base (neutral) reactions, reduction reactions, oxidation reactions), and thermal treatments (pyrolysis/gasification) [10]. To modify lignocellulose, it is of great interest to replace organic solvents with environmentally safe biological treatments that have opened up new options for biorefineries and economic reasons.

Biological pretreatment is a promising technique as there is no inhibitor formation during the process, ecofriendly, and low-cost procedure [11, 12]. Biological pretreatment methods are usually performed by microorganisms, the usage of microbial consortium and fungus could produce potent ligninolytic and cellulolytic enzymes [13, 14]. Microorganisms have evolved the ability to change and access lignocellulosic biomass. Exploiting this capability provides a natural, low-input method of preparing biomass for biofuels operations. Natural modification and degradation, in particular of the lignin component, can minimize the severity needs of future thermochemical pretreatment stages. The type of microorganism plays a very important role in the efficiency of biological delignification and makes a positive contribution to environmental protection [15, 16]. White-rot fungi were considered to be the most efficient microorganisms for lignin decomposition in nature, which represent that a low-cost process been applied to ecofriendly alternative pretreatment or copretreatment methods [17–19]. Some soil filamentous fungus-like *Mycelia sterilia* could decompose the lignin of oat straw in a comparable degree with white-rot fungi [20]. Different from fungi, bacterial strains degrade lignocellulose in the way by tunneling into the interior cell walls or making stripy erosions in the microfibrils of cellulose and have extensive interactions for lignin degradation, which show potential to processing lignocellulosic waste biomass. Bacterial ligninolytic enzymes are actively involved in the degradation of phenols, diamines, aromatic amines, and other xenobiotic molecules [21, 22].

Thus, microbes are crucial in delignification, and further research is needed to find new, efficient microorganisms. Therefore, the current study's goal was to isolate, screen, and identify effective lignocellulolytic enzyme-producing microbes from diverse sources.

## 2. Materials and Methods

### 2.1. Culture Media and Conditions

For isolation, storage, and maintenance microbial culture, the following culture media were used in this study: (1) potato dextrose agar (PDA) medium (for fungi): obtained from Qingdao Hope Bio-Technology Co., Ltd. and (2) Luria-Bertani (LB) agar medium (for bacteria): purchased from Beijin Aoboxing Bio-Tech Co., Ltd. Differential medium: (1) 0.04% guaiacol-added maintenance medium [23]; (2) 0.01% aniline blue-added maintenance medium [24]; and (3) congo red-CMC medium: sodium carboxymethyl cellulose (CMC-Na) 2 g/L, KH_2_PO_4_ 1 g/L, MgSO_4_·7H_2_O 0.5 g/L, tryptone 1 g/L, Congo-Red 0.4 g/L, agar 20 g/L, pH 7 [25]. Liquid medium for enzyme assay: (1) basic liquid medium: LB/PDB medium; (2) 1% cellulose (CMC-Na/Avicel) added Mandels & Andreotti medium: KH_2_PO_4_ 2 g/L, (NH_4_)_2_ SO_4_ 1.4 g/L, MgSO_4_·7H_2_O 0.3 g/L, CaCl_2_ 0.3 g/L, FeSO_4_·7H_2_O 5.0 mg/L, MnSO_4_·H_2_O 1.6 mg/L, ZnSO_4_·7H_2_O 1.4 mg/L, CoCl_2_ 2 mg/L, peptone 1 g/L, yeast-extract 0.05 g/L, Tween-80 2 mL/L [26]. Medium for solid-state fermentation: 10 g corn stalks (20 mesh), 50 mL Mandels & Andreotti medium.

### 2.2. Sampling and Isolation Procedures

Soil collected from forests, surface humus, straws, wood, fresh herbivore manure, and edible mushrooms were used for the isolation of the potential microbes. Environmental samples such as soils, hummus, and wood samples were collected from the Shandong University of Technology; straws were collected from nearby fields of Shandong and neighboring provinces. Excrement of farm-raised gray rabbits from farms was collected as herbivore manure samples. For isolation of bacteria and fungi, the environmental samples were mixed with sterile distilled physiological saline at room temperature for 1 h with shaking at 180 rpm, and the water extract supernatant was serially diluted till 10^−7^ dilutions. 100 *μ*L aliquots from the dilution 10^−4^ to 10^−7^ were taken and spread onto plates of different types of isolation medium. All plates were incubated at 28 and 37°C for fungi and bacteria, respectively.

Macrofungi isolated from edible mushroom-stick and internal tissues were cut from primordium hypha blocks and transferred onto a PDA medium for further isolation. Microbial cultures of all above were monitored and purified in time by stapping inoculation/streaking and disc transfer method. Pure strains based on colony morphology were stored on a maintenance medium at 4°C for further experimentations.

### 2.3. Screening of Potential Isolates

The guaiacol and aniline blue indicators were used and supplemented in the differential medium for screening laccase-producing, lignin degradation-related peroxidases-producing (lignin peroxidases and manganese-dependent peroxidase) microbes, and congo red-CMC medium aid in detecting cellulases production by these microbes. Organisms with brown oxidation in 0.04% guaiacol and/or discoloration in 0.01% aniline blue were selected as potential strains. Discoloration of congo red reflects the cellulase activity of the selected strains. In the differential process, activated bacteria and fresh newly harvested spores of filamentous fungi were inoculated on a differential medium and basidiomycete fungi transfer onto the differential medium by 5 mm mycelial disk inoculation. Bacterium and fungus plates in screening were, respectively, cultivated at 37°C and 28°C and observed for the development of colored/decolorization reaction and were assessed on daily basis.

### 2.4. Extracellular Enzyme Assays

Positive strains of guaiacol brown oxidation and aniline blue decolorization were selected for enzyme activity detection. Bacteria were inoculated at 2% and grown at 37°C for 48 h at 180 rpm, and the fungi were inoculated with 10^−6^ mold spores/mL or 5 mm hyphae block per 5 mL (mushrooms or fungus difficulty in collecting spores) at 28°C for 7 days with shaking at 180 rpm. Cultures were filtrated through nylon fabric and then centrifuged at 10,000×g at 4°C for 10 min. The enzyme activities of all resultant supernatants were estimated. Laccase, lignin peroxidases, manganese-dependent peroxidase, CMCase, avicelase, and *β*-D-glucosidase activities were analyzed of all selected isolates. Laccase (Lac) activity was determined by oxidation of 2,2′-azino-bis-(3-ethylbenzthiazoline-6-sulfonate) (ABTS) method [27].

Assays were performed in a 3 mL mixture containing 2.7 mL 50 mM sodium acetate buffer (pH 5.0), 15 mM ABTS 200 *μ*L, and suitably diluted crude enzyme 100 *μ*L. The oxidation of ABTS was performed at room temperature by monitoring spectrophotometrically the change in absorbance at 420 nm. One unit of enzyme activity is defined as the amount of enzyme required to oxidize 1 *μ*moL ABTS/min using an ɛ420 value for oxidized ABTS of molar absorption coefficients 36,000 M^−1^ cm^−1^.

MnP activity was measured by the oxidation of Mn^2+^ [28]. 3 mL reaction mixture consists of 50 mM sodium lactate buffer (pH 4.5) 2.7 mL, 1.6 mM MnSO_4_ 100 *μ*L, suitably diluted crude enzyme 100 *μ*L, and activator 1.6 mM H_2_O_2_ 100 *μ*L; the reaction is initiated at room temperature by the addition of H_2_O_2_ and the increase in absorbance measured at 240 nm. One unit of enzyme activity is defined as the amount of enzyme required to form 1 *μ*moL of Mn^3+^/min using an ɛ240 value for Mn^3+^ of molar absorption coefficients 6,500 M^−1^ cm^−1^.

Lignin peroxidase (LiP) activity was measured as described by Tien and Kirk [29]. LiP activity was determined at room temperature in a 3 mL reaction mixture containing 2.24 mL 50 mM sodium tartrate buffer (pH 2.5), 10 mM veratryl alcohol 600 *μ*L, suitably diluted crude enzyme 100 *μ*L, and activator 20 mM H_2_O_2_ 60 *μ*L. The reaction is initiated by the addition of H_2_O_2_ and the increase in absorbance measured at 310 nm. One unit of enzyme activity is defined as the amount of enzyme required to form 1 *μ*moL of veratraldehyde/min using an ɛ310 value for veratraldehyde of molar absorption coefficients 9,300 M^−1^ cm^−1^.

CMCase reaction mixture containing 1 mL of appropriately diluted enzyme and 3 mL of 1% CMC-Na in 50 mM citric acid buffer (pH 4.8) was incubated at 50°C in water bath for 30 min and terminated by dinitrosalicylic acid (DNS) 3 mL, and after 5 min, was diluted with 25 mL dH_2_O [30]. Reducing sugar levels in the supernatant was determined at 540 nm [31]. Avicelase reaction mixture containing 1 mL crude enzyme and 3 mL of 1% avicel in 50 mM citric acid buffer (pH 4.8) was incubated at 50°C for 1 h and measured with the same method of CMCase [31]. CMCase and avicelase were calculated as *μ*moL reducing sugar released (U/g). The *β*-D-glucosidase activity was assayed with modified method of Kovács; the assay mixture contained 1 mL 5 mM 4-nitrophenyl-*β*-D-glucopyranoside (pNPG) in 0.05 M sodium acetate buffer (pH 6.0) and 100 *μ*L appropriately diluted enzyme solution; the mixture was incubated at 50°C for 10 min, terminated by 2 mL 1 M Na_2_CO_3_, and diluted with 10 mL with dH_2_O; the liberated p-nitrophenol (p-NP) level was determined at 405 nm [32]. The *β*-D-glucosidase activity was calculated as *μ*moL *ρ*-NP released per minute per g of compost (U/g). All assays above were performed in triplicate, and the data presented in the tables correspond to mean values with a standard errorless than 10%.

### 2.5. Selection and Identification of Isolates

According to the result of extracellular enzyme activity assays, the promising strains were selected for colony morphology, microscopic visualizations, and molecular identification followed by solid-state fermentation. The 16S rRNA genes of bacteria were amplified by PCR using primers 27F and 1492R and ITS genes of fungi using primers ITS-1F and ITS-4R for sequence analysis. Bacterial isolates were grown in the LB medium at 37°C for 24 h. The cultures were centrifuged at 10,000×g for 1 min, and the supernatant was removed. DNA extraction was performed using a TIANamp Bacteria DNA Kit (Tiangen Biotech Corporation, Beijing, China) according to the manufacturer's instructions. Fungal isolates were grown in the PDA medium at 28°C for 5-7 days, harvested hyphae collected by centrifugation through the suction filter, and rinsed with distilled water. DNA extraction was performed using a DNAquick Plant System (Tiangen Biotech Corporation, Beijing, China) according to the manufacturer's instructions. The amplified products were sent to the Sangon Biotechnology Co. Ltd., China, for sequencing. All the obtained sequences were deposited in the NCBI database with given accession numbers. The sequences were then aligned and compared by BLASTN available in (NCBI) GenBank to identify these closest phylogenetic relatives by sequence similarity searches. Molecular Evolutionary Genetics Analysis (MEGA 10) software was used to conduct sequence alignment and to help identify members of gene families.

### 2.6. Solid-State Fermentation (SSF) and Analysis

The solid-state fermentation was performed using corn stalks as agrowaste substrates. Bacteria and fungi were grown in the same way as submerging conditions; potential microbes were inoculated with 10 mL/flask and incubated for 30 days at 37°C (bacteria) and 28°C (fungi) under dark conditions. Culture biomass was collected, and their cell dry weight and enzyme activities were measured. 15 mL sodium phosphate buffer (pH 7.0) per gram of the wet substrate was added to 100 mL flasks for the enzyme assay analysis; above 80% wet substrate was dried at 45°C to reserved fermented sample for analysis of physical changes.

### 2.7. Scanning Electron Microscopy (SEM)

Scanning electron micrograph was used to observe and analyze physical changes by different types of tested microbes in the treated substrates. Images of the substrates were taken using a Thermo Scientific Apreo scanning electron microscope (SEM) (Quanta 250 FEG, FEI Co., Salt Lake, UT, USA). The dried samples to be analyzed were coated with a thin layer of gold for 30 sec, using SEM ion sputtering device and affixed to the sample holder. The acceleration voltage was 200 V-30 kV, and the current passed was 15 mA.

### 2.8. X-Ray Diffraction (XRD) Characterization

The changes in crystallinity of the samples before and after SSF were estimated by the XRD analysis of the substrates. Scanning was performed on a Bruker AXS X-ray diffractometer instrument set at 40 kV, 30 mA, The wavelength of the Cu/K*α* radiation source was 0.15418 nm, and the 2*θ* scan range was from 3° to 45° with a step size of 0.02°/min. Crystallinity index (CrI%) was calculated according to the following Segal proposed equation: CrI% = (*I*_002_ − *I*_am_)/*I*_002_ × 100%, where CrI represents the relative degree of crystallinity (%), *I*_002_ is the intensity of crystal plane at the 002 peaks (at 2*θ* = 22°), and *I*_am_ is the intensity at 2*θ* = 18°. The *I*_002_ peak corresponds to the crystalline fraction, and the *I*_am_ peak corresponds to the amorphous fraction [33].

## 3. Results and Discussion

### 3.1. Isolation and Preliminary Screening of Lignin-Degrading Strains

The present study is an effort towards an exploration of potent microbes for their selective delignification activity. While in our explorations, we have isolated and purified 105 morphologically different isolates from various samples. All the isolated strains were subjected to preliminary screening. Based on the formation of a clear visible zone around the colony on the solid media supplemented with the suitable specific indicators which demonstrated that isolates have ligninolytic or cellulolytic activity. Laccase-positive strains were identified by dark brown oxidation of guaiacol, and LiP/MnP active strains were identified by the decolorization of aniline blue. From the results of the preliminary screening process, 36 isolates showed a visually positive result in the guaiacol oxidation or aniline blue decolorization. Based on the morphological characteristics, these ligninolytic active isolates belonged to bacteria, basidiomycete fungi, and filamentous fungi present in Table 1. During our screening process, 15 bacteria, 7 basidiomycete fungi, and 5 lower fungi tested positive for laccase activity, and all these isolates were also positive for peroxidases activity. Rich supplements of specific substrates like phenolic substrates indicate secretion of ligninolytic enzymes was proven in other studies [34–36]. All the ligninolytic enzyme-positive strains tested for cellulases activity by congo red-CMC produced variable zones (CMC clearance) around their colony, and the decolorized zones of congo red-CMC demonstrated the ability to degrade CMC [37]. Cellulases play a crucial role in degrading the lignocellulosic biomass to release fermentable sugars. There is a requirement for enzymatic saccharification of natural lignocellulose, and when working with lignocellulosic substrates, the utilization of cellulose-degrading enzymes becomes critical [38]. Fungi contain most of the known wood-rot microorganisms. The ability of wood-decaying fungi, particularly white-rot and brown-rot fungi (WRFs and BRFs), to efficiently alter, degrade, and depolymerize important plant cell wall components has been extensively researched [39, 40]. In the present study, it was observed that fungi presented better levels of ligninolytic and cellulolytic activity than most bacteria; they were therefore considered key candidates. Among them, the highest ligninolytic scoring was demonstrated by 7 white-rot basidiomycete isolates that showed strong oxidation of guaiacol. Compared with basidiomycete isolates, the other filamentous isolates perform better activities on cellulases rather than ligninases, and almost all the bacteria isolates show limited efficiency on oxidation of guaiacol and aniline blue. It is well known that the degradation of lignin by bacteria are least effective than fungi, but the exploration of bacteria species for lignin depolymerization potentials are considered significant to industrialization of the biocatalytic/extracellular enzyme process [41].

### 3.2. Extracellular Enzyme Profiling

Different types of hydrolytic and oxidative enzymes were detected in cultures for lignocellulolytic enzyme production. The selected isolates were evaluated for extracellular enzyme profiling including laccase, LiP, MnP, CMCase, avicelase, and *β*-D-glucosidase activities in the liquid culture medium. Secretion of the extracellular enzyme plays a vital role in the lignocellulosic decaying process of biomass depolymerization and/or functionalization [42].

The isolates exhibited different levels of enzyme activity, and the enzyme activity profile of bacteria and fungi was, respectively, presented in Tables 2 and 3. All the tested isolates hardly adapt to CMC/MCC media as a single-carbon source, which resulted in quantifiable ligninolytic and cellulolytic activity in some cultures. To those isolates for which CMC/MCC was too difficult to hydrolyze and utilize in these conditions, it was hard to create an effective adjustment strategy on extracellular enzymes in nutritional deficiencies, as complex molecules are not easily utilized compared to simple monosaccharides like glucose [43]. The not detectable enzymatic activity of both bacteria and fungi has been not shown in Tables 2 and 3.

As shown in Table 2, bacterial strains A5, A10, A11, S2G3, S1P2, B0G2, and C5L2 showed Lac activity and A3, A5, A10, and A11 showed MnP activity, whereas, LiP activity is detected in most bacteria. The entire three major ligninolytic enzyme activities were recorded in the bacterial isolates A5, A10, and A11. Bacteria Lac level was not as good as fungi; however, the strains A5, A10, and S1P1 showed strong LiP activity of 18.28, 13.98, and 23.66 U/L, respectively, which was better than fungi. Bacterium coded as A5 contains both LiP and Lac level that was higher than the previously reported bacteria *B. subtilis* E3 (1.48 × 10^−5^ U/mL), which was suggested to be a cheap source of LiP for large-scale commercial production [44].

CMC/MMC are not good carbon sources for fungal isolates and do not grow well in these media. In the MMC medium, fungal hyphae were almost invisible and enzymes were undetectable. CMC also rendered some ligninolytic enzymes undetectable or inhibition of some cellulase. From Table 3, it was observed that fungi appeared to be a good producer of Lac and cellulases than bacteria in the basic PDB medium. Basidiomycete fungi showed maximum ligninolytic and cellulolytic enzyme level. From the results of Lac and MnP activity of basidiomycete isolates, it was observed that isolates RP, BP, and XZ showed activities of Lac, LiP, and MnP. The Lac is the most widely used ligninolytic enzyme, which can be varied applied to lignocellulose biorefineries, chemical modification, waste treatment, and stain decoloration [45–47]. The Lac activity of basidiomycete fungi isolates ranged from 61.11 to 802.78 U/L, and isolate HJ gets the highest activity. Lac reaction of these isolates is more active and sensitive than other extracellular enzymes, which is considered a potential value for the enzymatic project. The enzyme MnP are the important peroxidases, which play an important role during the initial stages of lignin degradation [48]. YH presents the best MnP activity of 19.83 U/L, which is a potential source for MnP production and phenolic compound oxidization.

The cellulases of fungi were not induced by cellulose as the only carbon source in cultivation media. It was concluded that there is strong growth inhibition in cellulose as a single-carbon condition, and avicel is not suitable for the growth and enzyme secretion of detected isolates. The difference of basidiomycetes between mediums indicating the ligninolytic performance is diverse with different substrates that the secretion of extracellular enzymes can be controlled with cultivated design and regulation in industrial sectors utilization [49]. CMCase, avicelase, and *β*-glucosidase assays were performed to understand the cellulolytic activity of these isolates; these cellulase activity levels reflect the ability of the isolates to be actively involved in the saccharification process of the delignified or cellulose substrates [50]. All fungi showed significant three major cellulase activities; some isolates observed cellulase induction of CMC cultivation. On the CMC medium, CMCase activities of B1P1, C4P1, and C5P1 slightly increased, and RP and XB perform detectable CMCase active. With CMC cultivation, *β*-glucosidase of C4P1 increased from 131.75 to 431.66 U/L, C5P1 increased from 263.1 to 343.23 U/L, *β*-glucosidase of R0P1 and avicelase of fungus XZ, B0P1, C0P2, C0G2, and C4P1 have small improvements. Compared with basidiomycetes, filamentous fungi have good secretion of cellulases but lower ligninolytic activity, and most of them more adaptable to the CMC medium.

Due to the lack of condition and inducer optimization study, the ligninolytic enzyme activities of these screened isolates are comparatively far from strains of deep research. Take Lac, for example, Zhang et al. report that enhanced *Trametes hirsuta* SSM-3 produce Lac 31777 U/L [51]. Rezaei et al. reported CuSO_4_ induced 4.8 U/mL Lac from *A. elongatus* [52]. Afreen et al. induce *Arthrospira maxima* with guaiacol on best carbon and nitrogen condition and obtained 54.671 U/mL maximum activity [53]. The enzyme activities of isolates reported in our study were much better than the same reported in strains like *Penicillium pinophilum* MCC 1049 (28.2 U/L) [54], *Stenotrophomonas maltophila* BIJ16 (208.23 U/L), and *Citrobacter freundii* LLJ16 (205.50 U/L) [55]. These isolates could be designated as the potential lignin degrader based on the enzyme quantification.

### 3.3. Molecular Identification of Microbial Strains

The cultural characteristics and morphological features results revealed that primary screened 36 isolates were identified as 22 bacteria, 7 basidiomycete fungi, and 7 other filamentous fungi. Among them, 4 bacteria and 10 fungi isolates were selected as potential candidates with high lignocellulose degradading activity, and they were further identified based on 16S rDNA and ITS sequences, colony morphology, and microscopic visualizations as shown in Table 4. Based on the 16S rDNA sequence, bacterial isolates were classified into genus *Bacillus* and *Chryseobacterium*, identified as members *B. subtilis*, *B. licheniformis*, and *Chryseobacterium gambrini*. *Bacillus* spp. are the most dominant bacterium used in the enzyme industry because of its ability to produce and secrete a rich amount of extracellular enzymes [56]. *Bacillus* such as *B. aryabhattai* and *B. altitudinis* were reported for cellulolytic activity studies [57, 58], but the bacterial laccases are relatively newer and their characterization followed to that of their counterparts in fungi. Laccase activities of *B. subtilis* and *B. licheniformis* were previously studied [59, 60]. Selected 3 filamentous fungi that were identified belong to *T. atroviride* and *Talaromyces funiculosus*. *Trichoderma* spp. are most studied regarding lignocellulosic degradation, and *T. atroviride* have been studied for the ligninolytic use on agrowastes composting [61]. There are few relevant studies of *Talaromyces*. In this study, basidiomycete isolates were identified as *P. citrinopileatus*, *P. ostreatus*, *P. djamor*, *P. florida*, *P. eryngii*, and *P. pulmonarius*. Most of the ligninolytic enzymes reported thus far are of fungal origin; in white-rot fungi, *Pleurotus* spp. are well known as fast-growing, and the biosafety has been studied extensively [62, 63].

**Figure 1:**
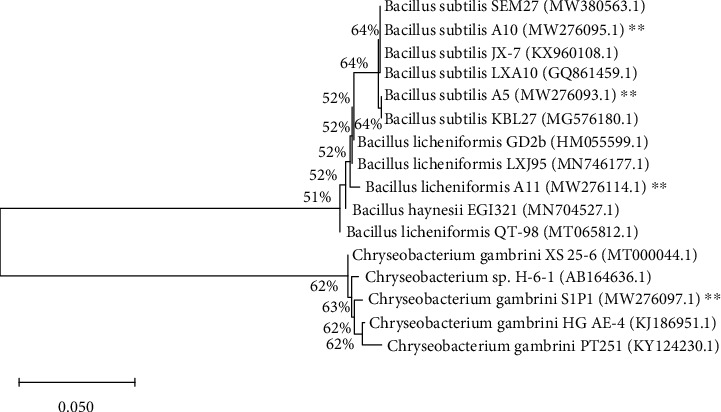
The neighbour-joining (NJ) phylogenetic tree based on 16S rRNA gene sequences of selected bacterial strains YH, HJ, HP, RP, BP, XB, and XZ with closely related strains accessed from the GenBank using BLASTN. These sequences were aligned using ClustalW. The NJ method was constructed using MEGA-X version 10.1.8. ^∗∗^Tested bacterial strains.

**Figure 2:**
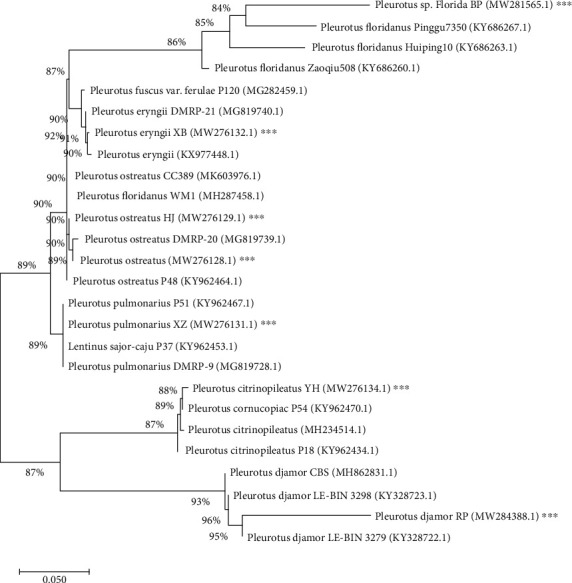
The neighbour-joining (NJ) phylogenetic tree based on 18S rRNA gene sequences of selected fungal strains A5, A10, A11, and S1P1 with closely related strains accessed from the GenBank using BLASTN (http://www.ncbi.nlm.nih.gov/blast/). These sequences were aligned using ClustalW. The NJ method was constructed using MEGA-X (Molecular Evolutionary Genetics Analysis; version 10.1.8). ^∗∗∗^Tested bacterial strains.

The phylogenetic tree also indicated the closest similarity to selected strains. The bacterial strains showed 99-100% sequence similarity with *B. subtilis* strain JCM 1465, *B. licheniformis* GD2b, and *C. gambrini* 5-1St1a, whereas the highly degradation activity selected fungal isolates showed 98-100% sequence similarity with *P. citrinopileatus* P54, *P. ostreatus* isolate CC389, and DMRP-20 and *P. djamor* LE-BIN 3279, *P. florida* Zaoqiu 508, *P. eryngii* DMRP-21, and *P. pulmonarius* P51. Based on the neighbour-joining method (Figures 1 and 2), the phenotypic and genomic data indicated that the selected bacterial and fungal strains represented strains of the genus *Bacillus* and *Pleurotus*.

### 3.4. Solid-State Fermentation (SSF)

SSF is not the main method in industrial-scale enzyme production; however, through SSF, ligninolytic enzyme production is higher and cost-effective rather than submerged fermentation (SmF) [64]. Lignocellulolytic enzyme production by different microbes is often enhanced in SSF cultivation for the reason it contains a growth substrate with a significant content of valuable nutrients as free mono- and disaccharides and organic acids [65]. To determine the efficiency of bacterial and fungal strain's individual potential of lignocellulose utilization in agro wastes, the complicated substrate such as corn stalks was chosen. The result of SSF is present in Table 5. The difference between substrate consumption in part reflects the efficiency of the biodegrading process. Some strains showed a good degradation efficiency in 30-day single-strain biotreatment; *C. gambrini* S1P2, *P. eryngii* XB, and *T. atroviride* C5P1 showed the ability to deplete more than 37% of materials, which is the comprehensive result of lignocellulolytic enzymatic reaction by these strains; shortened solid-state fermentation period is enough for the biodegrading effect.

The enzyme activity results presented in Table 5 clearly shows enzymes were still active in 30 days' fungal cultures, and there are advantages of corn stalk as a growth substrate for fungal lignocellulolytic enzyme production. The 6 enzyme activities were all detectable in isolates A5-, YH-, HJ-, HP-, and XB-treated substrates. HP and HJ present the highest laccase activities of 14.02 and 14.38 U/g, respectively. XB showed the best MnP activity of 2.81 U/g. All the species have shown cellulose-degrading enzyme activity that was necessary for enzymatic saccharification of complicated natural biomass. *T. atroviride* C5P1 showed the highest levels of both CMCase, *β*-glucosidase, and avicelase. Not the only cellulase function could get fast biodegradation, with the similar enzymatic performance like C5P1, isolates C4P1 are not well at solid-state fermentation, which may due to the differences of growth rate, adaptability, and enzymatic expression in the early fermentation. For the highest enzymatic activity related to growth, substrate and cultivation type, pH values, incubation temperatures, isolates are of different cultivation conditions to reach a maximal yield of enzymes [66]. Since there was almost no production of ligninolytic enzymes of filamentous fungi, the presence of a suitable incubation period was a determining factor for ligninolytic enzyme production. As the ligninolytic enzymes are produced as secondary metabolites in fungi, in most cases, the production is delayed, but early production was also observed in some fungi [67]. Elshafei et al. reported delayed Lac formation of 7.18 U/mg in *P. martensii* NRC 345 with production optima on the 26th day [68]. Mazumder et al. reported the maximum Lac production (3 × 10^5^ U/L) in SSF *P. ostreatus* at the 10th day, and the time can be reduced to 6 days [69]. In bacteria cases, the incubation time for optimum laccase production is very brief. In *Pseudomonas extremorientalis* BU118, it takes only 24 h [70], and in *B. tequilensis* SN4, it takes 96 h for [71]. That is the reason bacterial Lac activity is low/no detectable in this study. In this study, species from various origins were compared for their ability to produce a lignocellulolytic enzyme in SSF. The observed lignocellulolytic enzyme activity can only represent enzymatic characters of isolates' late period performance in long time SSF. The detectable activity of most lignocellulolytic enzymes of Pleurotus isolates indicates balance and long-acting enzymatic hydrolysis and oxidation consist in fermented materials; it means white-rot decay enzymes system of *Pleurotus* spp. are more stable on agrowaste substrates, as it was reported [72, 73]. As reported, agroindustrial wastes SSF with improvement; laccase activity was high up to2.90 × 10^5^ U/g in *T. giganteum* AGHP [74] and 1645 IU/g in *Bacillus* spp. MSK-01 [75].

### 3.5. Scanning electron Microscopy Analysis

**Figure 3:**
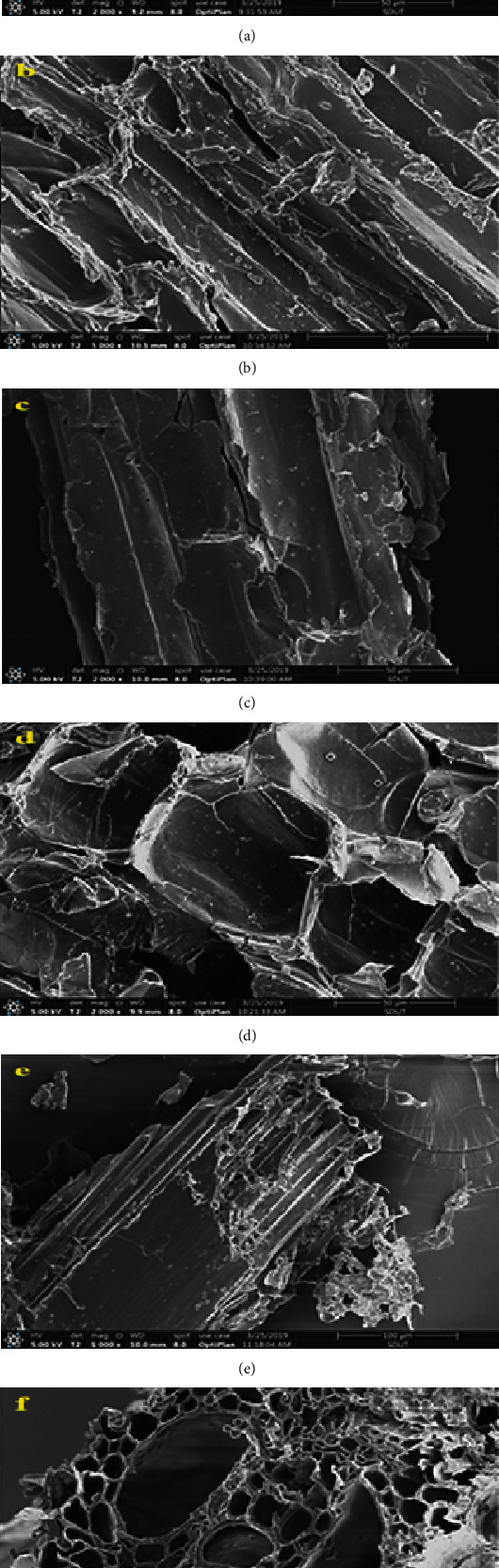
Scanning electron microscopy images of substrate: (a) untreated corn stalk, (b). *B. subtilis* A10-treated corn stalk, (c). *T. atroviride* C4P1-treated corn stalk, (d) *P. djamor* RP-treated corn stalk, (e). *T. atroviride* C5P1-treated corn stalk, and (f) *T. funiculosus* R0P2-treated corn stalk.

The SEM images of the untreated and treated substrates with a single isolate gave evidence of the physical changes that occurred during the treatment (Figure 3). In the case of untreated corn stalks, which had a compact fibrillary structure, the structures appear smooth and the ordered arrangements can be observed on the surface (Figure 3(a)).

A major improvement was observed after biopretreatments. The microfibers in the cell wall structure were completely disrupted and formed a new pattern with an expanded surface area. It appears that some biopretreatment generated a more conglomerate texture with a sponge-like structure. Bacteria can enter through the breaks of natural biomass structure and grow inside (Figure 3(b)). After being treated by bacteria, corn stalks did not show much change, and microorganism proliferation seems to not leave corrode marks on the smooth surface except the sharp edge being passivated. But the bioincising effect is much more visible on fungi-treated substrates (Figures 3(c)–3(f) ), the corn stalks treated by fungi showed disrupted surfaces and flaking out. The contact with the lignocellulolytic enzyme caused the surface to roughen and form densely corroded marks or holes. The external fibers are loosened during fungal treatment. On white-rot fungi-treated materials (Figure 3(d)), the microorganism erodes seriously; most of these surface erosions has perforated. Scanning of recognizable transfer passage tissue on corn stalks shows fungi hardly grow inside materials; the fungal growth decay of natural lignocellulose biomass mainly affects on outside region (Figure 3(f)). Comparing the waxy surfaces of corn stalks before/after pretreatment (Figures 3(a) and 3(e)), the microbial effect makes no difference on the surfaces from the wax-protected side.

### 3.6. X-Ray Diffraction (XRD) Analysis

The XRD patterns of the corn stalk materials mainly present two peaks, which are supposed to represent the typical cellulose I structure, namely, the amorphous region and crystalline region diffraction peak of cellulose at 16° and 22°, respectively [76]. The peak around 16° of the two peaks' combination indicates the presence of some small amount of cellulose II structure. Before and after the SSF pretreatment step of isolates, all the samples (untreated and pretreated) showed the typical XRD peaks of cellulose. There were no pronounced differences among the XRD profiles of samples, indicating that the biopretreatment cannot make a drastic change of the crystalline nature of corn stalk cellulose. It means the ordered structure of the crystalline region in their remaining cellulose was not disrupted by the contact with lignocellulolytic enzyme and microorganisms growth. After SSF, a significant variation in he diffraction pattern was observed in all SSF biopretreated samples: two peaks around 16° overlapped into one broad and weaker peak, and the sharp crystalline peaks around 22° transformed into a smooth and broadening peak; it means biopretreatment may change some angles around and hydrogen bond rearrangement *β*-glycosidic linkages of the crystalline region.  Table 6 presents the CrI values for the untreated and isolate-pretreated corn stalk materials. The effect of biopretreatment on the relative crystallinity biomass was different. CrI values increased after pretreatment that was observed in samples treated with isolates: *Pleurotus djamor* RP, *Pleurotus florida* BP, *Bacillus licheniformis* A11, and *Chryseobacterium gambrini* S1P2. In these samples, corresponds of both 18° and 22° was significantly decreased, but crystalline cellulose was still more recalcitrant than amorphous cellulose to microbial and enzymatic attack in bioprogress. It is a slight difference with identical XRD patterns that were documented in other pretreatment procedures which affect on cellulose harvesting and conversion [77–79]. According to our findings, most microbial pretreatment significantly increases cellulose CrI, which could be attributed to a selective disintegration of the amorphous sections of cellulose, which are more vulnerable to enzyme degradation. Despite prior results indicating a decrease in cellulose digestibility with increasing CrI of cellulose (on pure substrate), differences in enzymatic hydrolysis performance cannot be addressed solely on CrI measurements with these substrates. This is due to the fact that pretreatment biomass materials contain varying proportions of amorphous-like components, which affect not only enzymatic digestibility (for example, lignin and hemicellulose) but also the accuracy of CrI measurement using X-ray diffraction.

From the results of Table 3, the CrI value of pretreatment sample treated with *T. funiculosus* R0P2 showed decreased Crl value 56.35% as compared to untreated 64.55%. The decrease in the CrI by this fungus could be due to its conversion of crystalline cellulose into less ordered amorphous form, and its accumulation was also reflected in the broadening of the peak in the XRD spectrum, which was commonly observed in effective biopretreatment or enzymatic pretreatment of biomass [80, 81]. The decrease of determined crystallinity index of treated materials may also be due to substantial removal of lignin and hemicellulose [82, 83], but detected CrI values of biopretreatment materials in this study seems no directed relationship with cellulolytic or ligninolytic enzyme perform, similar with Moutta et al.'s study [84]. Our findings are consistent with prior research that found that chemical pretreatment alters the crystallinity of cellulose materials, but only when a significant amount of lignin is removed because delignification contributes to the enhancement of cellulose content in recovered materials [85, 86]. To date, little is known about how cellulose CrI changes during hydrolysis, and this change is highly dependent on the lignocellulosic substrates used as well as the mechanism of action of cellulases derived from various sources [87].

For XRD characterization of lignocellulosic materials before and after biopretreatment, the determined crystallinity index (CrI, %) is a fast and simple procedure for estimation of relative crystallinity; it is an important characteristic that makes the lignocellulosic network resistant to enzymatic reactions, which is often used as a measure of the relative amount of crystalline material present in a lignocellulosic sample reflect the change of susceptibility to enzymatic hydrolysis of substrates [88] and used to evaluate different biopretreatment types on the production of value-added bioconversion [89]. Microorganisms that can cause CrI values increasing can be used as a safety pretreatment instead of classic pretreatment in the cellulose industry to prepare enzymatic sensitive materials for various processes, while microorganisms of the opposite effect on CrI values intend to break down crystalline region efficiently in the lignocellulosic biodegradation; they can be used as a microbial element in mixed cultivate cofermentation or step fermentation system, which help create an economically applicable method on industrial scale biological reaction.

## 4. Conclusions

In the present study, 22 bacterial strains and 14 fungal strains with lignin-degrading ability were isolated based on guaiacol colorization and aniline blue decolorization methods. The production level of ligninolytic and cellulolytic enzymes in different medium conditions was compared. 4 bacteria, 7 basidiomycete fungi, and 3 filamentous fungi identified as species of genus *Bacillus*, *Chryseobacterium*, *Pleurotus*, *Trichoderma*, and *Talaromyces* were selected as potential lignocellulose pretreatment strains. Identification of such potent isolates from natural habitats is an ongoing process and is required for developing a sustainable, efficient, and economically feasible technology. Lignocellulolytic activities of fungal isolate have an enormous potential for utilization of lignocellulosic substrates, as they are the major group of microorganisms capable of synthesizing enzymes to degrade these substrates. The potent organism can be used for large-scale lignocellulolytic enzyme production and its use of treating various industrial effluents, especially in the lignocellulosic industry; these potential isolates were set up to study their bioconversion efficiency. These results encourage further investigation of ligninolytic and cellulolytic activities of isolated strains to evaluate their efficiency for possible industrial applications.

## Figures and Tables

**Table 1:** The preliminary screening results of selected lignin-degrading isolates.

Strain code		Origin of sample(s)	Guaiacol	Aniline blue	Congo red-CMC
Bacteria	A1	Soil (Shandong University of Technology)	−	+/−	++
	A3	Soil (Shandong University of Technology)	−	+/−	++
	A5	Soil (Shandong University of Technology)	+	+/−	++
	A6	Soil (Shandong University of Technology)	+/−	+/−	++
	A7	Soil (Shandong University of Technology)	−	+/−	++
	A8	Soil (Shandong University of Technology)	−	+/−	++
	A9	Soil (Shandong University of Technology)	−	+/−	++
	A10	Soil (Shandong University of Technology)	+/−	+/−	++
	A11	Soil (Shandong University of Technology)	+/−	+/−	+
	S2L2	Forest soil (Shandong University of Technology)	+/−	+/−	+
	S2L3	Forest soil (Shandong University of Technology)	+/−	+/−	++
	S0G1	Forest soil (Shandong University of Technology)	+	+/−	++
	S0G2	Forest soil (Shandong University of Technology)	−	+/−	++
	S2G2	Forest soil (Shandong University of Technology)	−	+/−	++
	S2G3	Forest soil (Shandong University of Technology)	+	+/−	++
	S1P2	Forest soil (Shandong University of Technology)	+	+/−	++
	M3L1	Forest humus (Shandong University of Technology)	+/−	+/−	+
	M3L2	Forest humus (Shandong University of Technology)	+/−	+/−	+
	M3L3	Forest humus (Shandong University of Technology)	+/−	+/−	+
	M3L4	Forest humus (Shandong University of Technology)	+/−	+/−	+
	B0G2	Decayed wood (Shandong University of Technology)	+	+/−	+
	C5L2	Corn stalks (Lianyungang, Jiangsu Province)	+	+/−	+

Basidiomycete Fungi	YH	Edible mushroom-stick (Linyi, Shandong Province)	+++	+	+/−
	HJ	Edible mushroom-stick (Linyi, Shandong Province)	+++	++	+/−
	HP	Edible mushroom-stick (Linyi, Shandong Province)	+++	++	+/−
	RP	Edible mushroom-stick (Linyi, Shandong Province)	+++	++	+/−
	BP	Edible mushroom-stick (Linyi, Shandong Province)	+++	++	+/−
	XB	Edible mushroom-stick (Linyi, Shandong Province)	+++	+	+/−
	XZ	Edible mushroom-stick (Linyi, Shandong Province)	+++	++	+/−

Filamentous fungi	B0P1	Decayed wood (Shandong University of Technology)	+/−	++	+++
	B1P1	Decayed wood (Shandong University of Technology)	−	++	+++
	C0P2	Corn stalks (Lianyungang, Jiangsu Province)	+/−	++	+++
	C0G2	Corn stalks (Lianyungang, Jiangsu Province)	+/−	++	+++
	C4P1	Corn stalks (Lianyungang, Jiangsu Province)	++	++	+++
	C5P1	Corn stalks (Lianyungang, Jiangsu Province)	++	++	+++
	R0P2	Gray rabbit manure (Zibo, Shandong Province)	−	++	+++

Note: screening of microbes was performed as described in Materials and Methods. Differential media of guaiacol, aniline blue, and congo red used for screening; Scoring: −: invisible; +/−: positive but barely visible; +: pale to moderately pale color; ++: moderately pale to strong color; +++: moderately strong to intense color.

**Table 2:** Enzymatic activities in cultures of bacterial isolates.

Bacterial strain(s)	Lac (U/L)	MnP (U/L)	LiP (U/L)	CMCase (U/L)	*β*-Glucosidase (U/L)	Avicelase (U/L)
A1	−	−	2.15	53.54	−	−
A3	−	3.42	−	9.22	−	7.69
A5	0.49	8.21	18.28	194.31	−	19.3
A10	0.29	5.47	13.98	210.86	−	24.82
A11	0.36	7.52	6.99	13.4	3.06	21.87
S2L2	−	−	2.15	24.43	−	15.59
S2L3	−	−	3.76	29.48	−	−
S2G3	0.14	−	2.15	37.94	4.21	−
S1P2	0.44	−	23.66	6.46	32.11	46.41
M3L2	−	−	2.15	54.87	10.79	17.68
M3L4	−	−	2.69	62.29	−	13.88
B0G2	0.15	−	1.08	128.2	14.94	20.25
C5L2	0.14	−	4.84	25.01	5.35	21.96

Note: −: not detectable under employed assay conditions.

**Table 3:** Enzymatic activities in cultures of fungal isolates.

Fungal strain(s)	Lac (U/L)		MnP (U/L)		LiP (U/L)		CMCase (U/L)		*β*-Glucosidase (U/L)		Avicelase (U/L)	
	PDB	CMC	PDB	CMC	PDB	CMC	PDB	CMC	PDB	CMC	PDB	CMC
YH	61.11	−	19.83	−	−	−	168.25	6.36	51.91	24.73	62.10	27.29
HJ	802.78	−	5.81	−	−	−	92.16	5.32	120.02	22.44	81.88	52.59
HP	452.78	−	1.37	−	−	−	240.53	7.69	62.21	23.01	87.59	42.32
RP	288.89	−	5.13	−	0.72	−	−	4.94	41.9	27.01	67.62	23.1
BP	447.22	−	4.79	−	0.72	−	134	4.94	41.04	10.13	61.15	42.13
XB	261.11	−	2.05	−	−	−	−	16.25	178.97	1.55	67.62	42.89
XZ	537.5	−	5.81	−	1.25	−	84.55	5.89	168.1	104.28	55.82	65.9
B0P1	0.56	−	−	−	−	−	17.4	7.03	457.41	184.12	49.54	65.71
B1P1	0.28	−	−	−	−	−	5.22	8.17	245.65	179.54	71.99	46.88
C0P2	3.19	−	−	−	−	−	56.01	22.34	209.3	205.3	21.96	84.36
C0G2	3.33	−	−	−	−	−	89.11	15.78	234.2	145.2	19.49	48.02
C4P1	5.38	−	−	−	−	−	17.4	60.48	131.75	431.66	47.45	63.62
C5P1	5.52	−	−	−	−	−	24.82	52.87	263.1	343.23	40.22	16.45
R0P2	6.67	−	−	−	−	−	232.92	16.16	2.12	46.76	149.22	59.25

Note: −: not detectable under employed assay conditions.

**Table 4:** Molecular identification of the cellulolytic selected isolates.

Code	Strain	Accession number	Strain of closest match
A5	*B. subtilis*	MW276093	*B. subtilis* JCM 1465
A10	*B. subtilis*	MW276095	*B. subtilis* JCM 1465
A11	*B. licheniformis*	MW276114	*B. licheniformis* GD2b
S1P1	*C. gambrini*	MW276097	*C. gambrini* 5-1St1a
YH	*P. citrinopileatus*	MW276134	*P. citrinopileatus* P54
HJ	*P. ostreatus*	MW276129	*P. ostreatus* CC389
HP	*P. ostreatus*	MW276128	*P. ostreatus* DMRP-20
RP	*P. djamor*	MW284388	*P. djamor* LE-BIN 3279
BP	*P. florida*	MW281565	*P. florida* Zaoqiu508
XB	*P. eryngii*	MW276132	*P. eryngii* DMRP-21
XZ	*P. pulmonarius*	MW276131	*P. pulmonarius* P51
C4P1	*T. atroviride*	MW276137	*T. atroviride* OUCMBI110146
C5P1	*T. atroviride*	MW276140	*T. atroviride* OUCMBI110146
R0P2	*T. funiculosus*	MW276141	*T. funiculosus* X33

**Table 5:** Substrate consumption and lignocellulolytic enzyme activity in corn stalk solid-state fermentation.

Code	Strain	Weight losses	Lac (U/g)	MnP (U/g)	LiP (U/g)	CMCase (U/g)	*β*-Glucosidase (U/g)	Avicelase (U/g)
A5	*B. subtilis*	29.59%	0.02	0.33	0.67	0.30	0.20	3.86
A10	*B. subtilis*	24.45%	−	0.50	0.63	0.35	0.08	4.22
A11	*B. licheniformis*	15.90%	−	0.05	0.42	0.31	0.16	4.20
S1P2	*C. gambrini*	37.57%	−	−	0.27	0.24	0.06	4.91
YH	*P. citrinopileatus*	16.29%	3.52	0.63	0.09	0.45	2.37	6.24
HJ	*P. ostreatus*	7.53%	14.02	0.81	0.03	0.30	1.50	3.97
HP	*P. ostreatus*	33.53%	14.38	0.79	0.09	0.24	1.92	2.35
RP	*P. djamor*	26.20%	1.82	0.05	−	0.23	0.29	3.26
BP	*P. florida*	15.67%	5.36	0.27	−	0.17	5.46	3.15
XB	*P. eryngii*	37.83%	4.57	2.81	0.40	0.31	4.44	5.59
XZ	*P. pulmonarius*	20.73%	11.82	1.27	−	0.33	3.13	6.79
C4P1	*T. atroviride*	17.80%	0.02	−	−	4.48	14.20	6.32
C5P1	*T. atroviride*	37.03%	−	−	−	4.61	24.83	6.39
R0P2	*T. funiculosus*	18.63%	−	−	−	1.66	1.35	4.80

Note: −: not detectable under employed assay conditions.

**Table 6:** The crystallinity indices of samples.

Substrate(s)	The crystallinity index (%)
Untreated corn stalks	64.55
Treated with *B. subtilis* A5	60.17
Treated with *B. subtilis* A10	62.71
Treated with *B. licheniformis* A11	72.83
Treated with *C. gambrini* S1P2	69.07
Treated with *P. citrinopileatus* YH	63.87
Treated with *P. ostreatus* HJ	61.06
Treated with *P. ostreatus* HP	63.04
Treated with *P. djamor* RP	68.18
Treated with *P. florida* BP	64.94
Treated with *P. eryngii* XB	61.29
Treated with *P. pulmonarius* XZ	63.83
Treated with *T. atroviride* C4P1	63.10
Treated with *T. atroviride* C5P1	61.60
Treated with *T. funiculosus* R0P2	56.35

## Data Availability

No data were used to support this study.
